# Evaluating concordance between government administrative data and externally collected data among high-volume government health facilities in Uttar Pradesh, India

**DOI:** 10.1080/16549716.2019.1619155

**Published:** 2019-06-04

**Authors:** Beth S. Phillips, Shreya Singhal, Shambhavi Mishra, Fnu Kajal, Sun Yu Cotter, May Sudhinaraset

**Affiliations:** a Institute of Global Health Sciences, University of California, San Francisco, CA, USA; b Community Empowerment Lab, Lucknow, India; c Department of Statistics, University of Lucknow, Lucknow, India; d National Health Mission, Lucknow, India; e Jonathan and Karin Fielding School of Public Health, University of California, Los Angeles, CA, USA

**Keywords:** Quality of care, external audit, government health system, incentives, India

## Abstract

**Background**: Globally, opportunities to validate government reports through external audits are rare, notably in India. A cross-sectional maternal health study in Uttar Pradesh, India’s most populous state, compares government administrative data and externally collected data on maternal health service indicators.

**Objectives**: Our study aims to determine the level of concordance between government-reported health facility data compared to externally collected health facility data on the same maternal healthcare quality indicators. Second, our study aims to explore whether the level of agreement between government administrative data versus the externally collected data differs by level of facility or by type of maternal health service.

**Methods**: Facility assessment surveys were administered to key health staff by government-hired enumerators from January 2017 to March 2017 at nearly 750 government health facilities across UP. The same survey was re-conducted by external data collectors from August 2017 to October 2017 at 40 of the same facilities. We conduct comparative analyses of the two datasets for agreement among the same measures of maternal healthcare quality.

**Results**: The findings indicate concordance between most indicators across government administrative data and externally collected health facility data. However, when stratified by facility-level or service type, results suggest significant over-reporting in the government administrative data on indicators that are incentivized. This finding is consistent across all levels of facilities; however, the most significant disparities appear at higher-level facilities, namely District Hospitals.

**Conclusion**: This study has a number of important programmatic and policy implications. Government administrative health data have the potential to be highly critical in informing large-scale quality improvements in maternal healthcare quality, but its credibility must be readily verifiable and accessible to politicians, researchers, funders, and most importantly, the public, to improve the overall health, patient experience, and well-being of women and newborns.

## Background

Globally, government health administrative data are a common source for deciding, funding, and evaluating national health programs. Open data platforms, news sources, and social media generally are making government or public sector data more accessible and consumable to the general public [1–4]. Meanwhile, those who access government data, such as researchers, politicians, consumers, and lay community members, are increasingly skeptical of these data sources [5,6]. While the quality of government data is often questionable, opportunities to validate government reports through external audits are also rare and typically constrained by timing, political instability and/or budgets [5,7]. This paper examines the results of a cross-sectional maternal health study by our team in Uttar Pradesh (UP), India’s most populous state, which involved review and audits of government facility data of government facility health data sources at select study sites.

Data sources typically defined as ‘government collected’ are those that are made or ordered by a government or government-controlled bodies and reported by government employees or workers, such as hospital administrative data, health department performance data or government health ministry findings. These sources enable public health researchers to explain overall health trends, develop standards for care, measure the impact of health policies or programs, to establish health performance benchmarks, or implement evidence-based policies [8–10]. Despite this ubiquitous utility and quantity of government data, limited research examines the reliability of these data. Previous research that examines the quality of government data are rare. These studies have focused on examining biases in administrative data [11,12] or to ascertain whether household survey data or government-reported data are more reliable for health systems strengthening [10,13,14]. For example, a recent study of a large clinical trial in the United States, Women Health Initiative (WHI), investigated the concordance between outcomes included in Medicare claims and those identified in the WHI protocol for cardiovascular events requiring hospitalizations [11]. While the authors posit limitations related to administrative barriers that may impact reporting, they argue that the general agreement found between Medicare claims and WHI data demonstrates an important step toward building evidence-based medicine within existing resources.

In other parts of the world, studies have compared household or community-level survey results to government administrative data and found more questionable concordance results [6,14–16]. A recent study from East Africa examines under-reporting and over-reporting in immunization coverage in Kenya by comparing household vaccination survey results to government administrative data [16]. The authors found that government data tended to misrepresent service use when tied to pay-for-performance initiatives. Specifically, the authors found that government data under-reported immunization coverage compared to household surveys. Consequently, donor funds went towards purchasing more vaccines than necessary, which ultimately wasted valuable resources [16].

India offers a unique context to examine the validity of government administrative data because the Government of India (GoI) routinely uses health facility data to inform national benchmarks and service provision guidelines. Of the few studies from India that examine government health data and its validity, all point to a paucity of comprehensive data sources on routinely collected and verifiable health data [14,15]. Morton, et. al. (2016) examine claims data from Rashtriya Swasthya Bima Yojana (RSBY), India’s biggest government-sponsored health insurance scheme, in one district of Orissa state, to determine health-care cost-effectiveness and government data quality. They conclude, however, that their analyses offer limited applicability for guiding health system strengthening in the state and informing other health-care settings due to lack of robust benchmarks for clinical quality in India. As a result, the reliability and generalizability of the district’s government insurance claim data remain questionable [15]. Similar to another health planning readiness study in peri-urban Kenya [16], a recent readiness assessment to implement Health Technology Assessment across India also finds differences between administrative data and household survey data [14]. Their findings suggest that government reports typically over- or under-report certain statistics that are tied to performance measures or to financing schemes.

These misrepresentations of health data often produce unintended consequences. Commonly termed ‘perverse incentives,’ this concept is cited among experts in health systems and health-care policy to explain over-reporting of certain procedures or protocols that have higher reimbursable monetary value to the facility and/or the actual health provider [6]. In other words, providers are paid more, or their health facility receives more reimbursements from health insurance schemes, such as government-sponsored insurance, when more patients get these ‘incentivized’ services. These incentives, however, may inadvertently or perversely jeopardize patient well-being and negatively affect health outcomes [6]. Recent reports [17] document high rates of cesarean section (C-section) which is the most common major surgical intervention in the world [18]. C-sections can be life-saving at 10% across a population and are typically reimbursed at higher amounts than vaginal deliveries [19]. When C-sections are unwarranted, these operations can also lead to an increased chance of obstetric and neonatal complications [19]. Sandefur and Glassman (2014) argue that such incentives often ‘perversely’ impact patient well-being, jeopardize government and funder mutual trust, and limit public expenditure efficiency by exaggerating progress on development or public health indicators [6]. Nearly 15 years ago, the GoI instituted *Janani Suraksha Yojana* (JSY) [20]. This program incentivizes women as well as health providers and facilities with cash transfers to have specific maternal health services done at a government health facility. The 2017 GoI’s Record of Proceedings (UP RoP) yearly budget outlines the specific amounts patients and providers receive for institutional vaginal births, C-section births, sterilization, Intrauterine Contraceptive Device (IUCD) and Postpartum IUCD (PPIUCD) insertions [21]. The line item amounts for C-sections and sterilizations are incentivized at nearly three times the amount for vaginal births and less invasive family planning services, such as birth control pills or Depo-Provera. Recent evidence suggests that these incentivized maternal health and family planning services under JSY may inadvertently undermine the Government’s aim to lower maternal mortality and morbidities and increase long-acting contraception use in India [22].

It is therefore critical to examine the quality of government self-reported health facility data. This paper reveals key consistencies and highlights striking discrepancies between government administrative health data and externally collected data on maternal health service indicators by at the same public health facilities in Uttar Pradesh, India. We hypothesize that government health facilities will over-report on incentivized Maternal Child Health (MCH) procedures, such as institutional deliveries, number of C-sections, sterilizations, IUCD/PPIUCD insertions, and staff numbers while underreport on other maternal health service indicators, such as number of essential obstetric medicines and post-natal beds, as compared to externally collected health facility data.

## Methods

This ancillary research study is part of the Quality-Plus (Q+) Study, which aims to understand the drivers of maternal health-care quality in high-volume, public maternity facilities in Uttar Pradesh (UP). High volume facilities are defined as those government health centers reporting 200 or more deliveries every month over the previous year. To create a representative sample of 40 high volume, health facilities across the State (including tertiary hospitals, maternity clinics, and community health centers), our team first developed a facility assessment survey to measure maternal health-care service performance. Due to study resources (including budget, research personnel, and timeline) and competing priorities, the Q+ study was limited to 40 high volume facilities in Uttar Pradesh. While the Ministry of Health and Welfare collects data from state and district health entities through its India Health Management Information System (IHMIS), this portal was not regularly updated [23]. Secondly, we noticed that the HMIS data for UP suffered many discrepancies and/or missing data in some programs, including family planning and immunizations. Therefore, we developed the Q+ facility assessment survey. It combines specific structural and process maternal health service indicators used and validated by previous researchers to measure maternal healthcare quality [24–26], including facility demographics, available procedures and services, types of patients, medicine and vaccine inventories, sanitation and hygiene, medical equipment, rooms and beds, and human resources. Questions asked for specific numbers or the availability of items/services currently at the facility. If permission was granted by the interviewee, enumerators looked at facility logs or physical storage units to confirm the availability of these supplies and recorded expiration dates of medication and vaccines. For questions related to a number of patients for specific services, such as deliveries, sterilizations, or IUCD/PPIUCD insertions, interviewees were asked to report the total patient number for the previous three months. For example, for surveys administered in February 2017, enumerators asked for the total number of patients who had IUCD/PPIUCDs inserted in January 2017, December 2016, and November 2016.

### Survey administration

This facility assessment survey was translated from English to Hindi and piloted at a government hospital in Lucknow, India in December 2016. From the pilot, we made minor updates to translations, re-structured certain questions, and re-organized the flow of the survey to reduce redundancies. Between January 2017 and March 2017, government enumerators, contracted and trained by the National Health Mission (NHM) Quality Assurance (QA) to conduct QA activities, conducted the Q+ facility assessment survey at 10 health facilities with highest patient loads in each of UP’s 75 districts (N = 750). They interviewed key government facility staff such as Management Officers, Chief Medical Officers, and/or Pharmacist Assistants. The government hired enumerators also conducted direct observations and review of facility records as question instructions indicated.

**Figure 1.**
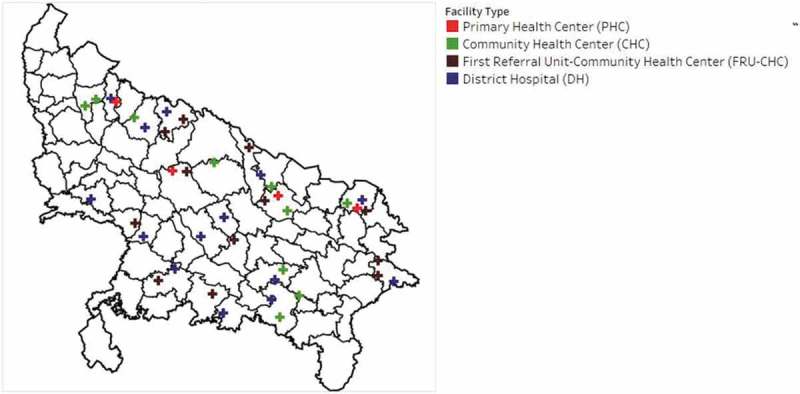
Map of Q+ study sites by facility type.

Following survey completion, data were used to select the Q+ study sites. Following analyses conducted by Nesbit, et al. [27] on the Service Provision Assessment (SPA) data, a nationally representative data source on the quality of care in over 30 countries [28,29], maternal health service indicators were summed to overall ‘maternal health service performance’ scores per facility. We ranked facilities according to their performance score. Then, we stratified all by geography and by facility-type to purposefully select a generally representative spread of 40 high-volume government health facilities in UP, as displayed in the study map (Figure 1).

During the site selection process and data review, the study team consulted with the local state health ministry and decided to validate these government-collected facility results through an external audit using the same Q+ facility assessment survey. Along with the original domains included in the Q+ facility assessment survey, daily monitoring indicators on beds, availability of food, ambulance, basic sanitation, and hygiene services were also assessed during our team’s external audit. This tool was administered in all 40 Q+ study facilities by locally hired, trained, non-government-employed data collectors, during August 2017-October 2017. The same interviewing and data collection techniques used by the government enumerators were employed, with training conducted by the joint study team. Ethical clearance for this research was provided by the ethics review boards of the authors’ respective institutions.

### Analyses of data

We compared facility assessment survey results from the government administrative data collection and the external Q+ data collection to evaluate the concordance between the two datasets. Of the 52 questions collected, we selected those most related to clinical quality, infrastructure, and maternal health outcomes. Procedures, such as deliveries and IUCD/PPIUCD insertions, are reported by average total number performed over the previous three months and grouped under ‘Reproductive health procedures and outcomes.’ Those questions related to infrastructure, such as staffing, essential drugs and vaccines, and medical equipment, are also averaged across all facilities’ totals. We termed these items ‘Facility Indicators.’ Using simple summary statistics, we compared these individual questions by examining the absolute difference and direction of the difference between their means. We used t-tests to see if these differences were statistically significant. Following this analysis, we identified and examined outliers at the facility-level through scatter plots on all indicators. Subsequently, we stratified by facility-type to assess the level of concordance within each level of care. Finally, we categorized services by incentivized versus non-incentivized services. We defined incentivized services following the GoI UP RoP 2017, as described previously [21].

We used Cohen’s Kappa scores to cross-validate the t-test results and to assess discordance between the individual level categorical government administrative data and the external audited results. The Cohen Kappa test was applied to each categorical variable and checked for concordance. The level of concordance was categorized into five categories based on the values of Cohen’s Kappa which are Poor (κΚ<0.0), Slight (κΚ = 0.0–0.20), Fair (κΚ = 0.21–0.40), Moderate (κΚ = 0.41–0.60), Substantial (κΚ = 0.61–0.80), and Almost Perfect (κΚ = 0.81–1.00).

All analyses were completed using Stata MP 15.0 [30].

## Results

From our comparative analyses of government administrative and the external facility datasets, we present the average frequencies and mean differences between the two datasets using both t-tests and the Cohen’s Kappa analyses, explore potential confounding, and assess concordance by facility-level of care and incentivized versus non-incentivized services.

Table 1 displays the government administrative reported means and external audit means of the main Maternal Health Service Indicators for maternal health. As described in our methods, these average frequencies are summarized across all facility types and organized by Reproductive Health Services or Facility Indicators, respectively. Overall, a majority of the indicators appear to match, and several differences between the mean frequencies reported by government vs external audit are statistically significant. Specifically, C-section (459.24) and sterilization clients (243.71) mean differences are statistically significant at p < 0.001 and p < 0.05, respectively. While facility indicators generally show less dramatic differences on average, government administrative results yield significantly more essential drugs for mothers and babies than our external audit results (p < 0.0001). Similarly, the government administrative data report 25 more beds in the post-natal ward (p < 0.001) than the audited data. Government administrative data also report more staff, especially clinical (n = 25) compared to the external audit (n = 17, p < 0.05).

**Table 1.** Maternal Health Service Indicators*.

Indicator name	Government administrative mean frequency (SD)	External collected mean frequency (SD)	*p*-Value
*Reproductive Health Procedures*			
Deliveries	973.55 (478.01)	914.03 (461.44)	0.06
C-sections	535.47 (575.79)	76.24 (155.66)	0.00
IUCD/PPIUCD^ insertions	394.75 (905.71)	268.24 (220.08)	0.37
Female Sterilization	287.32 (596.14)	43.61 (65.99)	0.01
*Facility Indicators*			
Basic medical equipment	7.88 (0.33)	7.93 (0.35)	0.32
Essential Drugs for mothers and infants	23.6 (6.74)	12.88 (4.46)	0.00
Vaccines	4.68 (2.27)	5.53 (1.09)	0.02
Functioning Toilets	9.18 (7.59)	9.18 (3.07)	0.46
Beds in Post Natal Ward	47.26 (42.78)	21.56 (14.76)	0.00
Estimated minutes to walk to safe drinking water	3.65 (6.06)	2.46 (1.91)	0.25
Clinical Staff	25.25 (23.56)	17.1 (8.43)	0.02
Non-Clinical Staff	7.18 (5.50)	6.38 (3.67)	0.36

* Reported over previous 3 months: Government collected: January-March 2017; External collected: May 2017-July 2017. ^ Intrauterine Contraceptive Device (IUCD) and Post-Partum IUCD (PPIUCD) insertions

We explored further by stratifying our results by level of care: District Women Hospital (DWH), First Referral Unit-Community Health Center (FRU-CHC), Community Health Center (CHC), and Primary Health Center (PHC) and by service type (incentivized vs non-incentivized). This dual-stratification, as displayed in Table 2, reveals that government administrative results tend to over-report services, most notably with incentivized services compared to externally collected results across all facilities. Reviewing the government-reported sterilization loads by facility type, DWHs report 20% more, CHCs and FRU-CHC nearly 9% more, and PHCs over 10% more cases compared to the audited data on sterilization cases. IUCD/PPIUCD insertions show less dramatic differences between government administrative and externally audited datasets. Interestingly, both PHCs and CHCs slightly under-report IUCD/PPIUCD insertions as compared to audited results, though negative trend results are not significant.

**Table 2.** Mean differences of Maternal Health Service Indicators, Stratified by Level of Care and Incentive Status*.

Facility Type	District Women Hospital (*n* = 14)	First Referral Unit-Community Health Center (*n* = 12)	Community Health Center (*n* = 10)	Primary Health Center (*n* = 4)
*Incentivized Services: mean difference (p-value)*				
C-sections	505.31 (0.01)	304.82 (0.01)	570.40 (0.04)	456.25 (0.32)
IUCD/PPIUCD insertions	22.43 (0.74)	426.58 (0.37)	−34.70 (0.36)	−6.25 (0.54)
Female Sterilization	374.43 (0.17)	179.50 (0.00)	132.00 (0.03)	202.25 (0.06)
*Non-incentivized Services: mean difference (p-value)*				
Monthly Deliveries	81.00 (0.27)	29.08 (0.44)	82.56 (0.08)	12.00 (0.91)
Essential Drugs for mothers and infants	11.07 (0.00)	9.00 (0.00)	11.60 (0.00)	12.50 (0.02)
Beds in Post Natal Ward	50.77 (0.00)	13.75 (0.00)	13.10 (0.01)	11.50 (0.23)
Clinical Staff	10.71 (0.22)	5.92 (0.21)	9.00 (0.11)	3.75 (0.35)

* Reported over previous 3 months: Government collected: Nov 2016-Feb 2017; External collected: May 2017-July 2017

**Figure 2.**
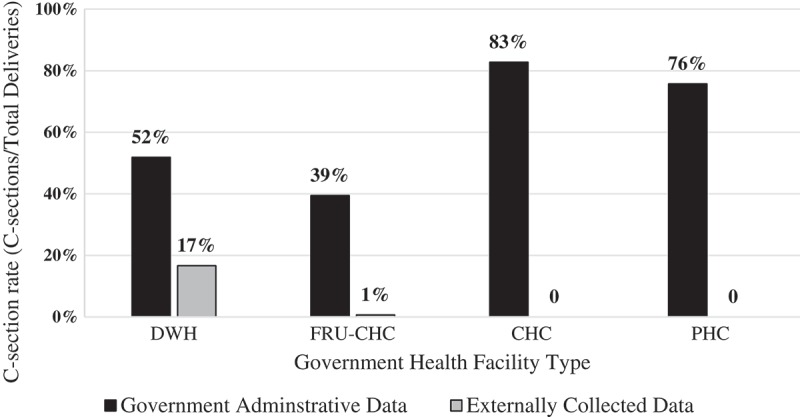
C-section rate by facility type.

Additional discrepancies exist between maternal health service indicators found in the government administrative data versus external audit data. For example, C-sections are heavily incentivized in India, and we find over 55% of the deliveries are C-sections in the government reported data compared to a C-section rate of 8% in audited data (Figure 2). Stratification of C-sections results by facility level of care reveals much higher C-sections across all facility types in the government administrative data versus the audited data. Specifically, according to the external audit findings, no C-sections occur at lower-level facilities (PHC and CHCs) which is expected since they are not equipped for these procedures, despite government data reporting C-sections. However, our stratified analyses also reveal that the FRU-CHCs where laboring women are first sent after visiting their local PHC or CHC are also performing very few C-sections according to the external audit (less than 5 C-sections reported over 3 months across all 12 FRU-CHC facilities). This finding dramatically contradicts the government administrative reported data. The audited data by level of care show that only DWHs perform C-sections, though also much fewer (n = 505.31, p < 0.001) than reported in the government administrative data.

Stratified results for non-incentivized services also reveal differences by level of care between the two datasets (Table 2). However, the discordance between government data and external audit data is much less compared to incentivized services, and especially minor among the monthly delivery loads. However, slightly larger differences are suggested among key infrastructure, medicine, and staffing indicators. For example, beds in post-natal wards and essential drugs for mothers and infants appear over-reported across all facilities. The largest discrepancies are found in the 14 DWHs with over 50 more beds and 10 more staff reported on average than actually found during the external audits (p < 0.001). There also seems to be a trend that clinical staff numbers are over-reported in PHCs, CHCs, FRU-CHCs, and DWHs by the government data sources.

To further assess concordance between the government reported and externally audited health facility data we performed the Cohen Kappa test on all categorical individual indicators. Table 3 presents only those agreement results that were significant by individual item. Concordance ranged from ‘slight’ to ‘fair’ on items related to the availability of essential medicines and vaccines for mother, while those scores for items, such as specialist doctor and pathologist available in facility, all show fair agreement levels. These results cross-validate the t-test overall scores presented above and further support the finding that the non-incentivized services show strong concordance across government reported health data and externally audited facility health data in UP.

**Table 3.** Cohen Kappa Results for Categorical Facility Indicators.

Indicators	Cohen’s Kappa Score	Quality of Agreement
*Medicines*		
Sodium Chloride	0.13	slight
Calcium Gluconate	0.28	fair
Gentamicin for mothers	0.30	fair
Metronidazole	0.26	fair
Misoprostol	0.19	slight
Azithromycin	0.26	fair
Nifedipine	0.30	fair
Zinc Tablets	0.35	fair
Domperidone	0.13	slight
*Vaccines*		
Measles vaccine	0.30	fair
Pentavalent vaccine	0.30	fair
*Staffing*		
Specialist doctors in facility	0.21	fair
Pathologist in facility	0.25	fair

## Discussion

While this study found high concordance between most maternal health-care indicators across government administrative data and externally collected data, the results suggest significant over-reporting by government sources on indicators that are incentivized at the facility-level or provider-level. In line with our original hypotheses, C-sections and sterilizations were particularly over-reported in government administrative data. Potential explanations for differences in reporting include the structure of India’s national health system that incentivizes health providers for certain types of procedures or outcomes, such as assisted deliveries, family planning services, and completed female sterilizations [21]. Incentives that are tied to performance whether in economics, education or health, are typically associated with little or no quality improvement and often with inaccurate data [31–33]. It is also important to note that larger discrepancies occurred at higher-level facilities – namely district hospitals – compared to other facilities. Potential reasons for this could include higher funding needs for district hospitals given the higher client volume. It is also possible that district hospitals may have less capacity for thorough record keeping compared to smaller hospitals, and therefore, results in differential reporting by government reporting and external auditing.

Governments around the world are increasingly driven by consumers and policy-makers to make government data publicly available, accessible, and reliable [2–4,34]. Identifying and understanding the gaps and limitations of government health data contribute to the GoI’s objective toward health systems strengthening and making verifiable, routinely collected health data the gold standard to achieve GoI’s goal toward universal health coverage in the achievement of Sustainable Development Goals [35]. India increasingly relies on its own internally generated funding and data collection sources to gather health data (moving away from reliance on large-scale donors or research institutes). Additional measures to safeguard data quality are essential [14]. Such safeguards could also improve overall health systems operations, patient wellbeing and health outcomes, and public confidence in government health data, by helping to avoid perverse incentives where certain medical procedures are tied to higher reimbursable values by government and/or private health insurance programs.

The proper use of validated public health facility data presents promising avenues to evaluate healthcare efficiency and effectiveness and to provide updated health information to policymakers in a cost-effective and publicly accessible and credible manner [13]. Even basic steps can be made to enhance India’s RSBY insurance scheme’s ability to track the quality of care, uncover low quality and learn from high quality, and thereby improve the overall facility-based provision of health care in India [15]. The GoI is actively working to address issues around ‘perverse incentives’ by applying a multi-strategic approach that involves government-sponsored health insurance schemes. In September 2018, for example, the GoI instituted a national health insurance protection program in UP entitled ‘Ayushman Bharat Yojana’ (ABY) to provide health insurance to 500 million Indians with an ancillary government entity, named NITI-Aayog, to perform external audits bimonthly [35]. ABY provides coverage up to 50,000 INR rupees (roughly USD 7000) per poor family per year for secondary and tertiary care hospitalization. To date, more than 14 million beneficiaries have already been admitted and received treatment under this scheme [36].

In terms of the limitations of this study, the sample size is relatively small. While the facility-reported data were collected across 727 facilities (as described in Figure 1), due to resource constraints and the parent study design, only 40 sites completed a Q+ facility assessment survey by an external data collection team. To ameliorate this potential limitation, the 40 study sites were chosen to represent geographic variation as well as the level of care across the state. We examined how these 40 sites compared to the remaining high-volume facilities not included (n = 206) and found no significant differences in the facilities that were and were not included in our study. Second, the different timing of the questionnaire could change responses naturally. This seasonality could bias the discordance results, especially with regard to childbirths, a well-established, worldwide example of seasonality [37,38]. However, as our analysis demonstrated, vaginal delivery loads did not show significant discordance between the government administrative data and the external data collection. Lastly, surveys were administered by different enumerators allowing for potential method administration variability, and respondents were not necessarily the same for each survey, though all survey respondents were facility-based, government-hired health providers. Future studies should combine observational data with facility staff reports and interviews conducted by external data collectors.

This study still holds a number of important maternal health service policy implications. First, when designing studies, determining funding decisions and allocating resources, governments, donors, and researchers should all be critical of reported health service data that are incentivized at provide or facility level. This study demonstrates that indicators such as C-section rates and female sterilizations are highly misreported, particularly by lower-level facilities. Allocating resources to facilities that have a genuine need for services, medicines, and equipment will lead to higher efficiency and equity in healthcare. Second, programs should set up standardized monitoring systems across all health facilities in India. This data system would include integrating audited data such as research-collected data, as well as routine administrative data, such as public insurance claims data. Importantly, training for implementation of these data collection systems should be rigorous, including standardized training for data collectors and government officials who are reporting on data. This training should also be tailored towards the level of care and stressing accurate reporting of all health indicators. Quality checks for data monitoring systems, like those common at many research institutions, should also be built into existing government data collection mechanisms, with ongoing quality checks performed.

## Conclusion

This study highlights key similarities and striking discrepancies between government administrative health data and externally collected data from the same public health facilities in Uttar Pradesh on maternal health clinical quality. From a health systems research perspective, this study suggests that non-incentivized indicators may have higher validity for broader research questions. With rapid digital advancements changing the global health landscape of how we, whether as funders, politicians, or researchers, think about and use government data sources, the importance of verifiably credible government data cannot be overstated. These data have the potential to be highly critical in informing large-scale quality improvements to the healthcare system to ultimately improve the overall health, patient experience, and well-being of women and newborns.

## Paper context

Government administrative data have the potential to be highly critical in informing large-scale quality improvement programs in maternal health in India and globally. Our study findings from India suggest significant over-reporting in government administrative data on maternal health indicators that are incentivized, with the largest discordance at the highest-level facilities. The credibility of government health facility data must be readily verifiable and accessible to politicians, funders, and the public to ensure robust health policies.

## References

[1] Alexopoulos C, Zuiderwijk A, Charapabidis Y, et al Designing a second generation of open data platforms: integrating open data and social media In: Janssen M, Scholl HJ, Wimmer MA, et al editors. Electron. Gov. [Internet]. Berlin, Heidelberg: Springer Berlin Heidelberg; 2014 [cited 2018 725] p. 230–9. Available from: http://link.springer.com/10.1007/978-3-662-44426-9_19

[2] Attard J, Orlandi F, Scerri S, et al A systematic review of open government data initiatives. Gov Inf Q. 2015;32:399–418.

[3] Chattapadhyay S. Access and use of government data by research and advocacy organisations in India: a survey of (potential) open data ecosystem. Proc. 8th Int. Conf. Theory Pract. Electron. Gov. [Internet] New York (NY): ACM; 2014 [cited 2018 725] p. 361–364. Available from: http://doi.acm.org/10.1145/2691195.2691262

[4] Jetzek T, Avital M, Bjorn-Andersen N The value of open government data: a strategic analysis framework. 2012 p. 12 CBS Research Portal Logo: https://research.cbs.dk/en/publications/the-value-of-open-government-data-a-strategic-analysis-framework

[5] Hut-Mossel L, Welker G, Ahaus K, et al Understanding how and why audits work: protocol for a realist review of audit programmes to improve hospital care. BMJ Open [Internet] 2017 [cited 2018 108];7 Available from: https://www.ncbi.nlm.nih.gov/pmc/articles/PMC5541620/10.1136/bmjopen-2016-015121 PMC5541620 28615270

[6] Sandefur J, Glassman A The political economy of bad data: evidence from African survey & administrative statistics - working paper 373 [Internet]. Cent Glob Dev. 2014 [cited 2018 518] Available from: https://www.cgdev.org/publication/political-economy-bad-data-evidence-african-survey-administrative-statistics-working

[7] Masood A, Lodhi RN Factors affecting the success of government audits: a case study of Pakistan. Universe J Manage. 2015;3(2):52–62. DOI:10.13189/ujm.2015.030202

[8] Chalkidou K, Marquez P, Dhillon PK, et al Evidence-informed frameworks for cost-effective cancer care and prevention in low, middle, and high-income countries. Lancet Oncol. 2014;15:e119–e131. 24534293 10.1016/S1470-2045(13)70547-3

[9] Commission on Evidence-Based Policy Making Commission on evidence-based policymaking releases final report [Internet]. 2017 p. 1 Available from: https://www.cep.gov/news/sept6news.html

[10] OCED Strengthening health information infrastructure for health care quality governance: good practices, new opportunities and data privacy protection challenges [Internet]. Paris: OECD Publishing; 2013 [cited 2018 515].

[11] Anderson GL, Burns CJ, Larsen J, et al Use of administrative data to increase the practicality of clinical trials: insights from the women’s health initiative. Clin Trials Lond Engl. 2016;13:519–526. 10.1177/1740774516656579 PMC5059108 27365013

[12] Braham RA, Finch CF The reliability of team-based primary data collectors for the collection of exposure and protective equipment use data in community sport. Br J Sports Med. 2004;38:E15. 15273208 10.1136/bjsm.2002.004002 PMC1724874

[13] Davis P, Milne B, Parker K, et al Efficiency, effectiveness, equity (E3). Evaluating hospital performance in three dimensions. Health Policy. 2013;112:19–27. 23537468 10.1016/j.healthpol.2013.02.008

[14] Downey L, Rao N, Guinness L, et al Identification of publicly available data sources to inform the conduct of health technology assessment in India. F1000Res. 2018;7:245. 29770210 10.12688/f1000research.14041.1 PMC5930391

[15] Morton M, Nagpal S, Sadanandan R, et al India’s largest hospital insurance program faces challenges in using claims data to measure quality. Health Aff Proj Hope. 2016;35:1792–1799. 10.1377/hlthaff.2016.0588 PMC7473072 27702951

[16] Otieno CF, Kaseje D, Ochieng’ BM, et al Reliability of community health worker collected data for planning and policy in a peri-urban area of Kisumu, Kenya. J Community Health. 2012;37:48–53. 21769730 10.1007/s10900-011-9414-2 PMC3258391

[17] Lancet T Stemming the global caesarean section epidemic. Lancet. 2018;392:1279. 30322560 10.1016/S0140-6736(18)32394-8

[18] World Health Organization WHO statement on caesarean section rates. Human reproduction programme (HRP): research for impact. Available from: https://apps.who.int/iris/bitstream/handle/10665/161442/WHO_RHR_15.02_eng.pdf;jsessionid=C0795F80C382D0461C13C6532EBD6B4E?sequence=1

[19] Sandall J, Tribe RM, Avery L, et al Short-term and long-term effects of caesarean section on the health of women and children. Lancet. 2018;392:1349–1357. 30322585 10.1016/S0140-6736(18)31930-5

[20] Fadel SA, Ram U, Morris SK, et al Facility delivery, postnatal care and neonatal deaths in India: nationally-representative case-control studies. PLoS One. 2015;10:e0140448. 26479476 10.1371/journal.pone.0140448 PMC4610669

[21] National Health Mission Record of proceeding: Uttar Pradesh 2017-2018 [Internet]. Ministry of Health and Welfare, Government of India; 2017 [cited 2018 94] Available from: http://nhm.gov.in/nrhm-in-state/state-program-implementation-plans-pips/uttar-pradesh.html

[22] Singh S, Doyle P, Campbell OM, et al Referrals between public sector health institutions for women with obstetric high risk, complications, or emergencies in India - a systematic review. PLoS One. 2016;11:e0159793. 27486745 10.1371/journal.pone.0159793 PMC4972360

[23] Institute for Health Metrics and Evaluation India health management information system (HMIS) | GHDx. 2019 [cited 2019 54] Available from: http://ghdx.healthdata.org/series/india-health-management-information-system-hmis

[24] Donabedian A Evaluating the quality of medical care. Milbank Q. 2005;83:691–729. 16279964 10.1111/j.1468-0009.2005.00397.x PMC2690293

[25] Health Statistics and Information Systems Service availability and readiness assessment (SARA): an annual monitoring system for service delivery [Internet]. World Health Organization (WHO); 2015 [cited 2018 64] p. 178 Report No.: 2.2. Available from: https://www.who.int/healthinfo/systems/SARA_Reference_Manual_Full.pdf

[26] Hill S Putting the priorities first: medicines for maternal and child health. Bull World Health Organ. 2012;90:87. 10.2471/BLT.11.088658 PMC3314208 22461719

[27] Nesbitt RC, Lohela TJ, Manu A, et al Quality along the continuum: a health facility assessment of intrapartum and postnatal care in Ghana. Plos One. 2013;8:e81089. 24312265 10.1371/journal.pone.0081089 PMC3842335

[28] Sheffel A, Karp C, Creanga AA Use of service provision assessments and service availability and readiness assessments for monitoring quality of maternal and newborn health services in low-income and middle-income countries. BMJ Glob Health [Internet] 2018 [cited 2019 38];3 Available from: https://www.ncbi.nlm.nih.gov/pmc/articles/PMC6267320/10.1136/bmjgh-2018-001011 PMC6267320 30555726

[29] Demographic and Health Surveys Program SPA overview. DHS Program. 2018 [cited 2019 37] Available from: https://dhsprogram.com/What-We-Do/Survey-Types/SPA.cfm.

[30] Stata. Stata statistical software: release 12. College Station 755 (TX): StataCorp LP; 2015.

[31] Chace Z How perverse incentives drive up health care costs [Internet]. Planet Money. NPR; 2014 [cited 2018 64] Available from: https://www.npr.org/2014/01/16/262946913/how-perverse-incentives-drive-up-health-care-costs

[32] Weeks J “Perverse incentives” and the triple aim: overcoming the troubled path to economic integration for integrative medicine and health. Glob Adv Health Med. 2015;4:7–9. 10.7453/gahmj.2015.005 PMC4424912 25984397

[33] Palmer N, Mueller DH, Gilson L, et al Health financing to promote access in low income settings—how much do we know? Lancet. 2004;364:1365–1370. 15474141 10.1016/S0140-6736(04)17195-X

[34] Martin EG, Begany GM Opening government health data to the public: benefits, challenges, and lessons learned from early innovators. J Am Med Inform Assoc. 2017;24:345–351. 27497796 10.1093/jamia/ocw076 PMC7651893

[35] National Portal of India Ayushman Bharat - national health protection mission [Internet]. Gov. India. 2018 [cited 2018 917] Available from: http://www.pib.gov.in/PressReleaseIframePage.aspx?PRID=1518544.

[36] National Health Authority Everyday PM-Jay update. Government of India. 2019 [cited 2019 37] Available from: https://www.pmjay.gov.in.

[37] Bernard RP, Bhatt RV, Potts DM, et al Seasonality of birth in India. J Biosoc Sci. 1978;10:409–421. 721846 10.1017/s0021932000011901

[38] Lam DA, Miron JA Seasonality of births in human populations. Soc Biol. 1991;38:51–78. 1749967 10.1080/19485565.1991.9988772

